# Balloon compression technique using an echoendoscopic balloon tip to prevent bile leakage in endoscopic ultrasound-guided choledochoduodenostomy

**DOI:** 10.1055/a-2587-9470

**Published:** 2025-05-19

**Authors:** Takashi Koriyama, Shunsuke Omoto, Mamoru Takenaka, Michihito Kono, Akito Furuta, Taro Inoue, Wataru Ono

**Affiliations:** 1 13737 Departments of Gastroenterology, Kishiwada Tokushukai Hospital, Kishiwada, Japan; 2 Departments of Gastroenterology, Kobe Tokushukai Hospital, Kobe, Japan, Kishiwada, Japan; 3 326473 Department of Gastroenterology and Hepatology, Kindai University Faculty of Medicine, Osaka-Sayama, Japan

Endoscopic ultrasound-guided biliary drainage (EUS-BD) is an alternative treatment for patients with malignant biliary obstruction or those with failed endoscopic retrograde cholangiopancreatography (ERCP) 1 2 3 4 . Endoscopic ultrasound-guided choledochoduodenostomy (EUS-CDS), a specific type of EUS-BD, can cause complications, such as bile peritonitis 5 . Previous studies have reported bile leakage in 2.8% of cases and peritonitis in 1.4% of EUS-CDS procedures 2 . In EUS-guided hepaticogastrostomy, a longer length of the liver parenchyma is associated with a reduced risk of bile leakage 3 . In contrast, EUS-CDS lacks surrounding organ support, potentially increasing the risk of peritonitis if bile leakage occurs ( Fig. 1 ). We developed a novel technique using balloon compression at the fistula site with an echoendoscope balloon tip to prevent bile leakage-induced peritonitis ( Video 1 ).

**Fig. 1:**
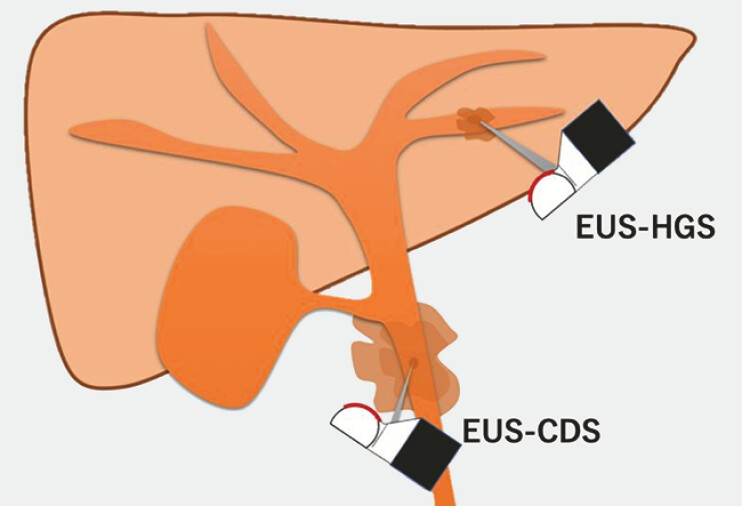
The liver parenchyma surrounding the bile duct prevents bile leakage in endoscopic ultrasound guided-hepaticogastrostomy (EUS-HGS), whereas endoscopic ultrasound guided-choledochoduodenostomy (EUS-CDS) has a higher risk of bile leakage because of the absence of surrounding organ protection.

During endoscopic ultrasound-guided choledochoduodenostomy, balloon compression at the fistula site using the echoendoscope tip before stent placement may prevent bile leakage-associated complications.Video 1

A 70-year-old woman presented with pancreatic cancer-caused obstructive jaundice. Pre-procedural computed tomography (CT) revealed a dilated common bile duct ( Fig. 2 ). ERCP was unsuccessful because of tumor invasion of the papilla. Therefore, EUS-CDS was performed as an alternative intervention. Using an echoendoscope (GF-UCT260; Olympus Medical Systems), the dilated common bile duct was visualized from the duodenal bulb. An EZ Shot 3 Plus 19G needle (Olympus Medical Systems) was used for puncture, and a 0.025-inch guidewire was successfully inserted. The puncture site was subsequently dilated to 4 mm using REN (Kaneka Medix Corporation).

**Fig. 2:**
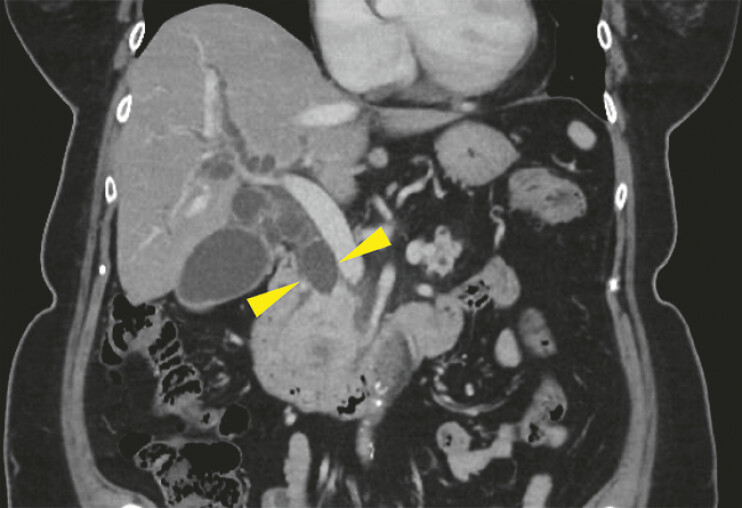
Pre-procedural computed tomography scan demonstrating a dilated common bile duct (yellow arrowhead).

Following fistula dilation, to prevent potential bile leakage, the balloon at the tip of the echoendoscope was inflated to compress the puncture site before metal stent insertion ( Fig. 3 ). Subsequently, a self-expandable metallic stent (Niti-S EUS-BD system; Century Medical) was placed. Post-procedural CT confirmed the absence of bile leakage ( Fig. 4 ). Fistula site compression using the echoendoscope balloon tip after fistula dilation and before metal stent placement may reduce bile leakage complications in EUS-CDS.

Endoscopy_UCTN_Code_TTT_1AS_2AG

**Fig. 3:**
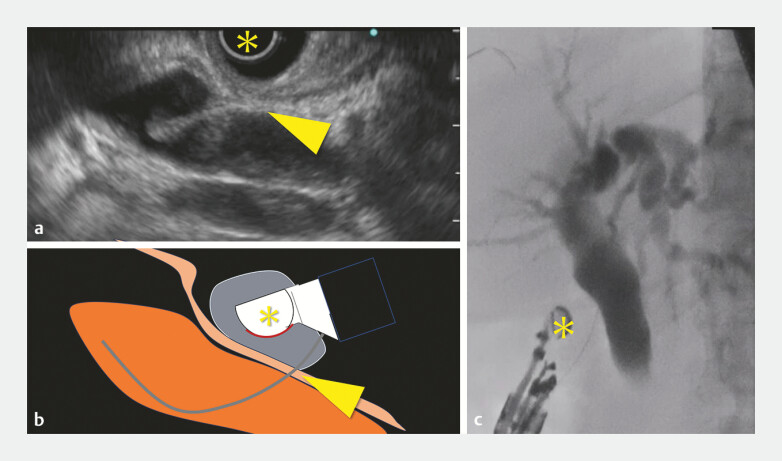
**a** Endoscopic image demonstrating the inflated balloon tip (yellow asterisk) compressing the fistula site (yellow arrowhead) following dilation. **b** Schematic representation of the balloon compression technique demonstrating the spatial relationship between the echoendoscope, balloon tip, and fistula. **c** Cholangiography demonstrating bile duct compression using a balloon tip.

**Fig. 4:**
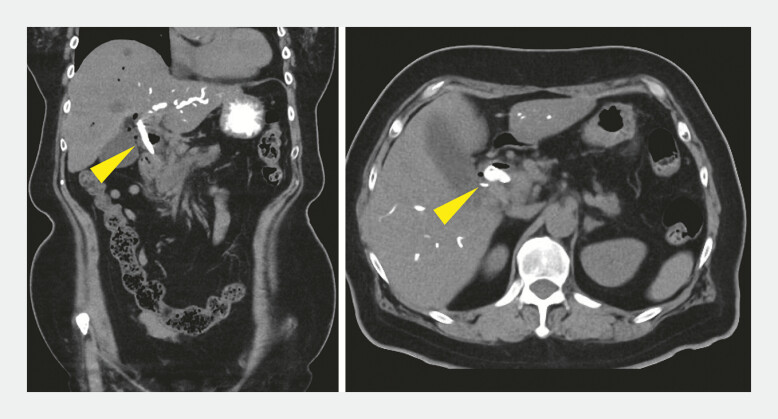
Post-procedure computed tomography scan demonstrating a properly positioned self-expandable metallic stent (yellow arrowhead) without any bile leakage.

## Notes

Endoscopy E-Videos https://eref.thieme.de/e-videos
*E-Videos* is an open access online section of the journal *Endoscopy* , reporting on interesting cases and new techniques in gastroenterological endoscopy. All papers include a high-quality video and are published with a Creative Commons CC-BY license. Endoscopy E-Videos qualify for HINARI discounts and waivers and eligibility is automatically checked during the submission process. We grant 100% waivers to articles whose corresponding authors are based in Group A countries and 50% waivers to those who are based in Group B countries as classified by Research4Life (see: https://www.research4life.org/access/eligibility/ ). This section has its own submission website at https://mc.manuscriptcentral.com/e-videos .
